# The Islet Estrogen Receptor-α Is Induced by Hyperglycemia and Protects Against Oxidative Stress-Induced Insulin-Deficient Diabetes

**DOI:** 10.1371/journal.pone.0087941

**Published:** 2014-02-03

**Authors:** Gamze Kilic, Ana I. Alvarez-Mercado, Bader Zarrouki, Darren Opland, Chong Wee Liew, Laura C. Alonso, Martin G. Myers, Jean-Christophe Jonas, Vincent Poitout, Rohit N. Kulkarni, Franck Mauvais-Jarvis

**Affiliations:** 1 Department of Medicine, Division of Endocrinology, Metabolism and Molecular Medicine, Northwestern University Feinberg School of Medicine, Chicago, Illinois, United States of America; 2 Department of Medicine, Division of Endocrinology and Metabolism, Tulane University Health Sciences Center, School of Medicine, New Orleans, LA, United States of America; 3 Montreal Diabetes Research Center, CRCHUM and Department of Medicine, University of Montréal, Montréal, QC, Canada; 4 Division of Metabolism, Endocrinology and Diabetes, Department of Internal Medicine, University of Michigan, Ann Arbor, Michigan, United States of America; 5 Section of Islet Cell Biology and Regenerative Medicine, Joslin Diabetes Center and Harvard Medical School, Boston, Massachusetts, United States of America; 6 Department of Medicine, Division of Diabetes, University of Massachusetts Medical School, Worcester, Massachusetts, United States of America; 7 Pole of Endocrinology, Diabetes and Nutrition, Institute of Clinical and Experimental Research, Catholic University of Louvain, Brussels, Belgium; Universidad Miguel Hernández de Elche, Spain

## Abstract

The female steroid, 17β-estradiol (E2), is important for pancreatic β-cell function and acts via at least three estrogen receptors (ER), ERα, ERβ, and the G-protein coupled ER (GPER). Using a pancreas-specific ERα knockout mouse generated using the Cre-lox-P system and a Pdx1-Cre transgenic line (PERαKO^−/−^), we previously reported that islet ERα suppresses islet glucolipotoxicity and prevents β-cell dysfunction induced by high fat feeding. We also showed that E2 acts via ERα to prevent β-cell apoptosis *in vivo*. However, the contribution of the islet ERα to β-cell survival *in vivo*, without the contribution of ERα in other tissues is still unclear. Using the PERαKO^−/−^ mouse, we show that ERα mRNA expression is only decreased by 20% in the arcuate nucleus of the hypothalamus, without a parallel decrease in the VMH, making it a reliable model of pancreas-specific ERα elimination. Following exposure to alloxan-induced oxidative stress *in vivo*, female and male PERαKO^−/−^ mice exhibited a predisposition to β-cell destruction and insulin deficient diabetes. In male PERαKO^−/−^ mice, exposure to E2 partially prevented alloxan-induced β-cell destruction and diabetes. ERα mRNA expression was induced by hyperglycemia *in vivo* in islets from young mice as well as in cultured rat islets. The induction of ERα mRNA by hyperglycemia was retained in insulin receptor-deficient β-cells, demonstrating independence from direct insulin regulation. These findings suggest that induction of ERα expression acts to naturally protect β-cells against oxidative injury.

## Introduction

The female steroid, 17β-estradiol (E2), is important for pancreatic β-cell function in mammals [1]–[4]. E2 acts through at least three estrogen receptor(ER)s in β-cells, ERα ER β and the G-protein coupled ER (GPER). These ERs are expressed in rodent and human β -cells in both sexes, where they exhibit a predominant extranuclear localization [3], [5]. The islet ERα is important for enhancing insulin biosynthesis *in vivo* via an extranuclear ERα-dependent mechanism that amplifies the effect of glucose in stimulating the insulin gene promoter [6], [7]. The islet ERα also suppresses excess *de novo* lipogenesis, which prevents glucolipotoxic β-cell failure in rodent models of type 2 diabetes (T2D) [8]. E2 also acts as a survival hormone that prevents β-cell apoptosis *in vivo* in both sexes at physiological concentrations. This protection is lost in mice globally deficient in ERα [9]. In cultured mouse and human islets, E2 protection is mediated mainly via ERα and GPER, and it protects from diabetes-associated injury resulting from oxidative stress and pro-inflammatory cytokines [5], [9]-[11]. Further, during pancreatic islet transplantation, use of an ERα-selective agonist enhances human islet graft survival, thus protecting islet functional mass [12]. Overall, global expression of ERα is necessary for islet survival in mice, and pharmacological activation of ERα protects islet survival in culture and following *in vivo* treatment. Nonetheless, the direct and singular impact of ERα in islet β-cells on islet survival *in vivo* –without contribution from the effects of ERα action in other tissues–has not been addressed. In this study we used the PERαKO^−/−^ mouse to examine the role of islet ERα in islet survival from alloxan induced-oxidative stress *in vivo*.

## Materials and Methods

### Generation of mutant mice and animal care

Pancreas specific ERα knockout mice were generated using the Cre-lox-P system and a Pdx1-Cre transgenic line (PERαKO^−/−^) as previously described [7]. Pdx1-Cre mice were bred onto the cre-inducible Rosa26-LacZ line at the University of Michigan. Animal had free access to food and water. They were kept on a 12-h light/dark cycle. All animal experiments were approved by Northwestern University or University of Michigan Institutional Animal Care and Use Committee.

### Induction of experimental diabetes and tissue collection

Diabetes was induced in 10–12 week-old female and male mice by a single intraperitoneal (IP) injection of 150 mg/kg of alloxan (ALX) (2,4,5,6-Tetraoxypyrimidine) (Sigma-Aldrich) freshly prepared in sterile cold saline (0.9%). Mice ERαlox^+/+^ were used as control for PERαKO^−/−^ mice. Blood glucose was measured every 48 h after ALX injection using One Touch Ultra Glucose Monitor (Lifescan). At day 11 after ALX injection, mice were killed and blood and pancreata were collected.

### *In vivo* drug administration

17β-Estradiol (4 µg/25 g); Tocris Biosciences) and vehicle (10% ethanol and 90% sesame oil) were administered subcutaneously (s.c.) twice daily for two days.

### Pancreas insulin concentration

Tails of the pancreata were collected, weighed, and homogenized in acid/ethanol. Then, pancreas homogenates were centrifuged, and supernatants were used to measure pancreas insulin concentration by radioimmunoassay (Linco) as described [7].

### Plasma insulin concentrations

Plasma insulin concentrations were measured by ELISA (Millipore).

### Pancreas immunohistochemistry

Deparaffinized pancreatic sections (5 µm) were blocked for 30 min with blocking solution (20% Fetal Bovine Serum + 2% Roche Blocking Reagent). Sections were incubated overnight with primary antibodies and 1–2 h with secondary antibodies at room temperature with the following primary antibodies: guinea pig anti-human insulin (1∶1000; Linco Research), rabbit anti-glucagon (1∶1000, Linco Research), rat anti-mouse CD31 (1∶400; BD Biosciences). Secondary antibodies FITC-conjugated donkey anti-guinea pig, CY3-conjugated donkey anti-rabbit, AMCA-conjugated donkey anti-guinea pig, and CY3-conjugated goat anti-rat (Jackson ImmunoResearch Laboratories) were used at concentrations recommended by the manufacturer. The nuclei were stained with DAPI (Invitrogen, Molecular Probes). Images were obtained with either Nikon Eclipse E400 microscope or Tissue Genostics Tissue/Cell High Throughput Imaging and Analysis System at Northwestern University Cell Imaging Facility.

### Brain immunohistochemistry

Perfusion and immunohistochemistry were performed as previously described [13]. Briefly, mice were anesthetized with a lethal dose of intraperitoneal pentobarbital (150 mg/kg) and transcardially perfused with sterile PBS and then either 4% paraformaldehyde or 10% formalin. Brains were removed, post-fixed overnight and dehydrated in a 30% sucrose solution. Following cryoprotection, brains were sectioned into 30 µm coronal slices, collected in four consecutive series and stored at −20°C until further use. For immunohistochemistry, brain sections were pretreated with ice-cold methanol, 0.3% glycine and 0.3% SDS before blocking. Sections were then incubated with primary antibodies either rabbit anti-ERα (1∶1000, Sigma) or goat anti-βGal (1∶1000, Biogenesis Ltd) overnight at 4°C. Detection of primary antibodies was done by either immunofluorescence using secondary antibodies anti-rabbit Alexa 488, anti-goat Alexa 568, both 1∶200 dilution (Invitrogen) or using the avidin –biotin/diaminobenzidine method (secondary antibody anti-rabbit –biotin, 1∶200 dilution).

### Cell counts and statistic

Stained sections were imaged using Leica microscope using 10X and 20X air objectives and processed using Adobe Photoshop CSII (Adobe Systems, San Jose, CA). Photoshop was only used to overlay matched images in different RGB channels such that dual-labeled cells would become apparent and could be quantified as described [13].

### Calculation of pancreatic β-cell mass

β-cell area was measured in insulin-stained 5 µm thick pancreatic sections. Three to four sections per tissue were randomly chosen for morphometric analysis. Insulin positive area was determined by using ImageJ 1.37v program. To calculate β-cell mass (mg), insulin positive area was divided by pancreas area and then multiplied by pancreas weight.

### Calculation of vessel density in islets

Blood vessel density was calculated by dividing the mouse-CD31-positive area by the insulin-positive islet area by using ImageJ 1.37v program.

### Rat model of glucolipotoxicity

Two-month and 6-month old male Wistar rats (Charles River, St.-Constant, QC), were maintained hyperglycemic and hyperlipedimic for 72 hours, following a co-infusion of 70% dextrose plus 20% Intralipid, compared to their 0,9% saline infused controls as described [14].

### Islets isolation

At the end of infusion, islets were isolated by pancreas collagenase digestion as described [14].

### Rat islets culture

Wistar rat islets were pre-cultured for a week in serum-free RPMI medium supplemented with 5 g/L BSA (37°C, humidified atmosphere containing 5% CO2). Islets were further cultured for 18 h or 1 week in the same medium containing 5, 10 or 30 mM glucose (medium was renewed every other day) [15].

### Mouse model of moderate hyperglycemia

To study ERα expression under mild hyperglycemia conditions, a 4 days glucose infusion in mice was performed as is described in [16]. Briefly, C57bl/6J male mice of 8–12 weeks-old and 20–25 g received a 4 days infusion of saline or 50% glucose. After this, mice were anesthetized and islets isolated by digestion with 1.7 ml/cc Collagenase P (Sigma) [9].

### βIRKO cell culture

We used insulin-secreting cell lines established from groups of βIRKO, and Lox control mice as was described previously [17], [18]. Cells were maintained at 37°C and 5% CO2 in Dulbecco's modified Eagle's medium (DMEM) containing 25 mM glucose, 10% fetal bovine serum, and penicillin and streptomycin. Experiments were performed using 80–90% confluent cells. Lox and βIRKO cells were seeded in 6-well plates and incubated for 24 h to recover. Cells were first washed with PBS before incubating in 16.7 or 33 mM glucose in DMEM containing 10% serum and penicillin and streptomycin for 3 days.

### Q- PCR

Total RNA was extracted using RNeasy® Micro kit (Qiagen, Valencia, CA) for islets or RNeasy® kit (Qiagen, Valencia, CA) for cells according to the manufacturer's protocols. cDNA was prepared from 1 µg of total RNA using the High Capacity cDNA Reverse Transcription Kit (Invitrogen) with random hexamer primers, according to the manufacturer's instructions Real-time PCR amplification of ERα and Tbp (TATA-box binding protein) cDNAs was carried out i on a CFX96 using iQ-SYBR green supermix (Bio-Rad, Hercules, CA). Results were normalized to TBP expression and expressed as arbitrary units. Primer sequences are the following: Rat islets: 5′ACCCTTCACCAATGACTCCTATG-3′ and 5′-TCAGCATTTCTGGCACGAAGT-3′for TBP and -5′AATTCTGACAATCGACGCCAG3′ and 5′-GTGCTTCAACATTCTCCCTCCTC-3′ for Erα. Mouse islets and cells: 5′-ACCCTTCACCAATGACTCCTATG-3′ and 5′-ATGATGACTGCAGCAAATCGC-3′ for TBP and 5′-GCTTCTCTTGGCCTGTACTT-3′ and 5′-CTCTCCCAGTTTCCACATCTT-3′ for ERα

### Statistical analysis

Data are presented as mean ± SEM unless otherwise stated. Data were analyzed by Student's *t test*. A value of *p*<0.05 was considered statistically significant.

## Results

### Recombination of ERα in hypothalamic neurons of PERαKO^−/−^ mice

**Figure 1:**
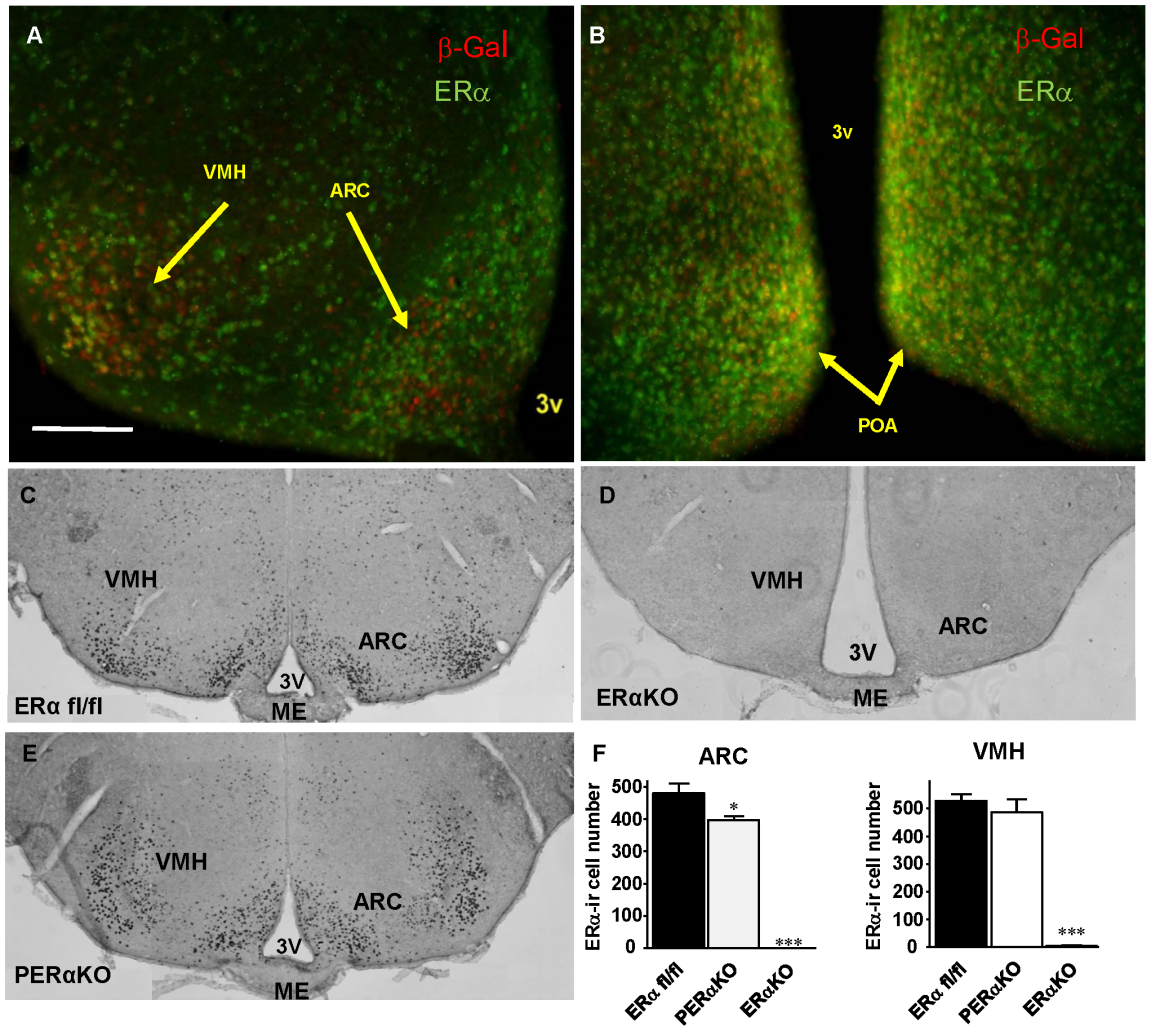
ERα expression in PERαKO^−/−^ hypothalamus. Pdx1-Cre/Lacz mouse and immunofluorescence in frontal brain sections from PERαKO^−/−^ mice. Pdx-1 expression (red) marked by beta galactosidase (β-gal) and ERα (green) show PDX-1 and ERα co-expression in the ventromedial hypothalamus (VMH), the preoptic area and arcuate nucleus (ARC) **(A–B)**. and quantification **(C)** Bar represents 250 µm. Representative images of immunohistochemical analisys showing ERα expression in frontal brain sections from ERα fl/fl, **(D)** ERαKO^−/−^ **(E)** and PERαKO^−/−^ **(F)** mice Pictures taken at 10X magnification and quantification **(G)**.

To investigate the role of pancreatic ERα on β-cell biology *in vivo*, we used PERαKO^−/−^ mice in which ERα was inactivated in all pancreatic lineages using a Pdx1-Cre transgenic mouse [7], [8]. Because Pdx1-Cre transgenic mice were reported to promote recombination in nutrient sensing hypothalamic neurons [19], we first sought to determine whether recombination of ERα occurs in the hypothalamus of PERαKO^−/−^ mice. Accordingly, using a transgenic Pdx1-Cre/LacZ mouse [19], we observed that Pdx1 is co-expressed with ERα in ∼26% of neurons of the ventromedial hypothalamus (VMH), ∼17% of neurons in the preoptic area (POA), and ∼15% of neurons of the arcuate nucleus (ARC) (Fig. 1A–B). Female PERαKO^−/−^ mice exhibited a 20% decrease in the number of ERα positive cells in the ARC, without a parallel decrease in the VMH (Fig. 1D–F). They also exhibited decreased fertility (data not shown), suggesting that ERαexpression was also decreased in the POA.

### No alteration in islet vascularization in absence of ERα

**Figure 2:**
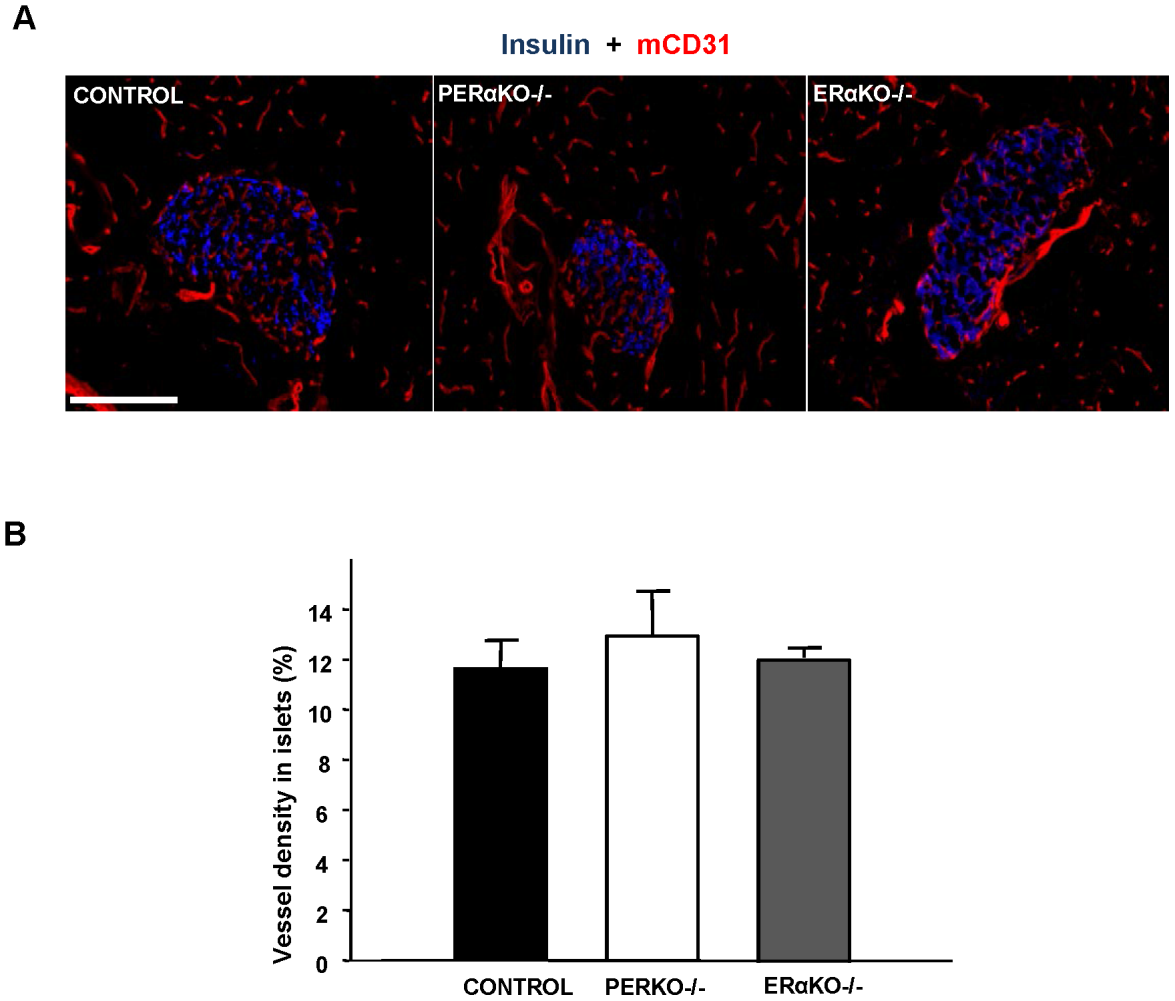
No difference in islet vascularization between ERαKO^−/−^, PERαKO^−/−^ and control female mice. (**A)** Representative sections showing immunofluorescence staining for insulin (blue) and mouse CD31 (red) positive cells in control, PERαKO^−/−^, and ERαKO−/− pancreata. (**B)** Quantification of islets vessel density. Values represent the mean±SEM, n = 3–4/group. Bar represents 100 µm.

E2 stimulates angiogenesis and promotes endothelial cell recovery after injury [13]–[17]. We previously observed that estrogens improve islet revascularization during islet transplantation [12]. Thus, prior to exploring islet predisposition to oxidative stress, we sought to determine whether islet vascularization was altered in female PERαKO^−/−^ mice. Because loss of ERα in β-cells or in endothelial cells can alter endothelial cell function via paracrine or endocrine mechanisms, respectively, we studied vascular density in PERαKO^−/−^ and mice globally deficient in ERα (ERαKO^−/−^). When we quantified the endothelial cell area in pancreas section using the mouse endothelial cell marker CD31, we observed no difference in islet vascularization among ERαKO^−/−^, PERαKO^−/−^ and female control mice (Fig. 2). The absence of islet vascular defects demonstrated that ERα was not essential for islet angiogenesis in mice.

### The absence of islet ERα predisposes to oxidative stress-induced diabetes in mice

**Figure 3:**
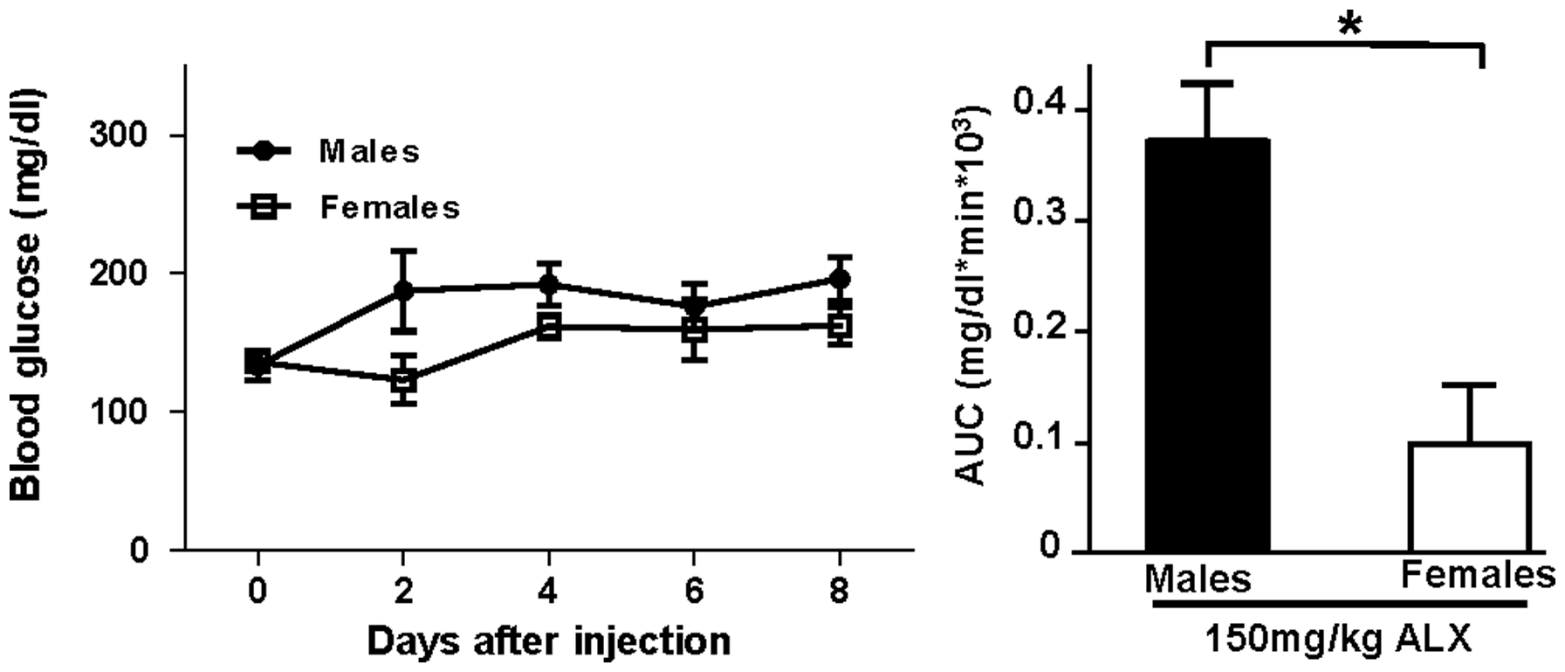
Gender dimorphism in alloxan sensitivity. Comparison of blood glucose values and area under the curve for glucose (AUC) above basal (T0) between male and female C57bl/6J wild type mice. Blood glucose was measured every 48 h for 8 days after injection of 150 mg/kg of alloxan (ALX). Values represent the mean±SEM, n = 4/group.*P<0.05.

**Figure 4:**
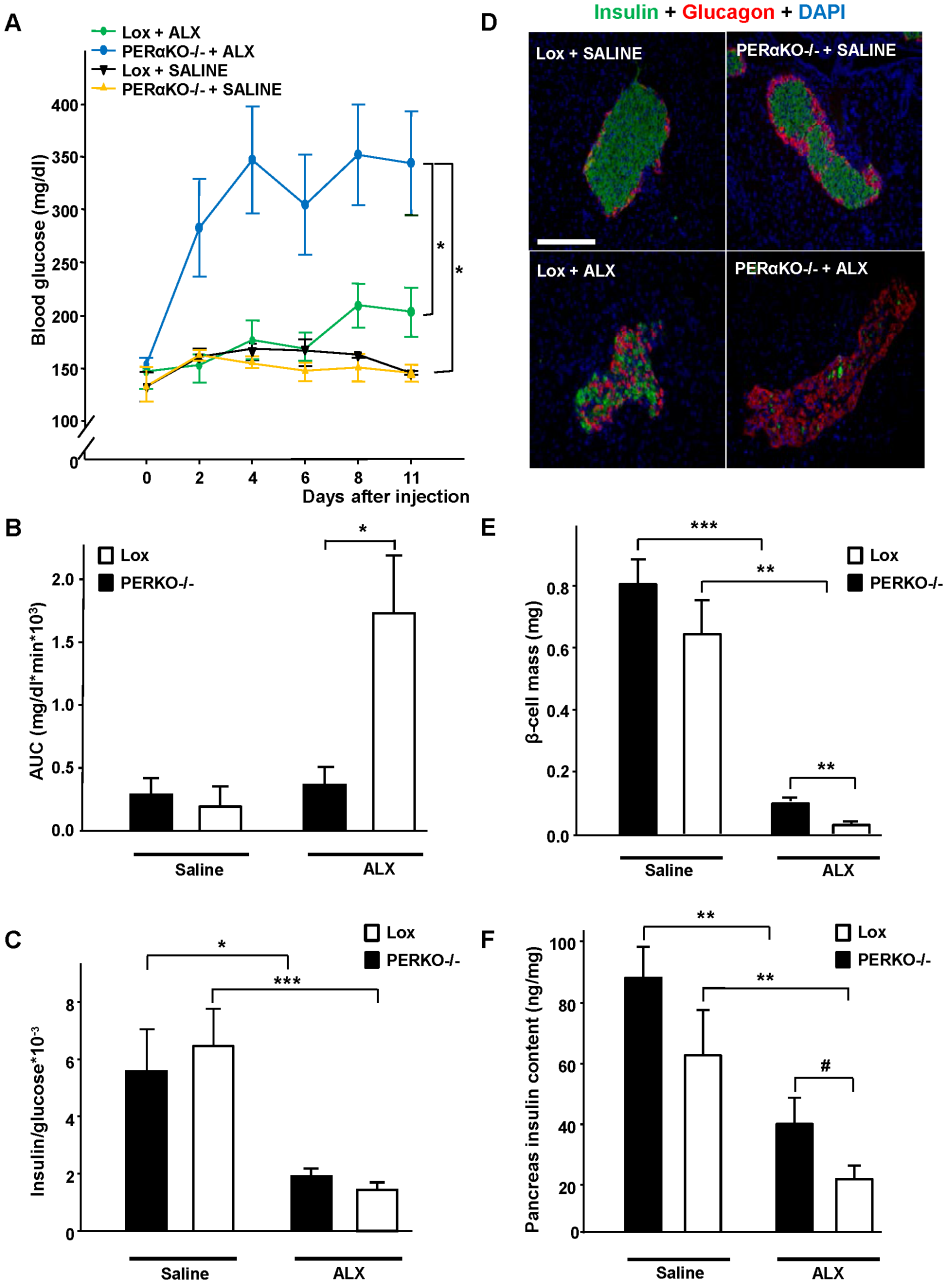
Female PERαKO^−/−^ mice are susceptible to ALX-induced diabetes. **(A)** Random-fed blood glucose from day 0 to day 11 after injection of either 150 mg/Kg of ALX or saline. **(B)** Corresponding area under the curve (AUC) for glucose. **(C)** Ratio of random-fed of insulin (ng/ml) and glucose (mg/dl) at day 11 was used as an index of insulin deficiency. **(D)** Representative sections showing immunofluorescent histochemical analysis of pancreas sections stained for insulin (green) and glucagon (red) in control ERαlox^+/+^ and PERαKO^−/−^ mice **(E)** β-cell mass quantification. **(F)** Pancreas insulin concentration 11 day after ALX injection. Values represent the mean±SEM, n = 4–13/group. *P<0.05, **P<0.001 ***P<0.01, Bar represents 100 µm.

We induced oxidative stress in β-cells *in vivo* using a single high-dose injection of alloxan (ALX; 150 mg/kg of body weight), which augments the generation of reactive oxygen species (ROS) in pancreatic islets [20]. We initially observed that female C57BL/6 mice were protected from ALX-induced diabetes (Fig. 3). Next, we induced oxidative stress in β-cells of PERαKO^−/−^ female mice. In basal conditions (time = 0, prior to ALX injection), control and PERαKO^−/−^ female mice displayed similar blood glucose (Fig. 4A–B) and insulin concentrations (data no shown). They also exhibited normal islet architecture, with insulin-producing β-cells in a central location and glucagon-producing α-cells at the periphery (Fig. 4D). PERαKO^−/−^ female mice showed a trend toward decreased pancreatic insulin concentration, an observation that was consistent with the known effect of ERα in stimulating insulin synthesis [6], [7]. Following exposure to ALX, control female mice showed relative protection compared to PERαKO^−/−^ female mice. Control female mice displayed only a minor increase in blood glucose despite hypoinsulinemia and an 87% decrease in β-cell mass and pancreatic insulin concentration (Fig. 4C–F). This finding was consistent with the fact that only 20% of β-cells are needed to maintain euglycemia [12]. In contrast, relative to controls, exposure of PERαKO^−/−^ female mice to ALX, induced marked hyperglycemia and insulin deficiency that resulted from a more severe β-cell destruction (97%) and decrease in pancreatic insulin concentrations (Fig. 4A–F). Thus, PERαKO^−/−^ female mice exhibited a predisposition to alloxan-induced β-cell destruction. Note that we did not observe differences in α-cell density between alloxan-injected control and PERαKO^−/−^ female mice.

**Figure 5:**
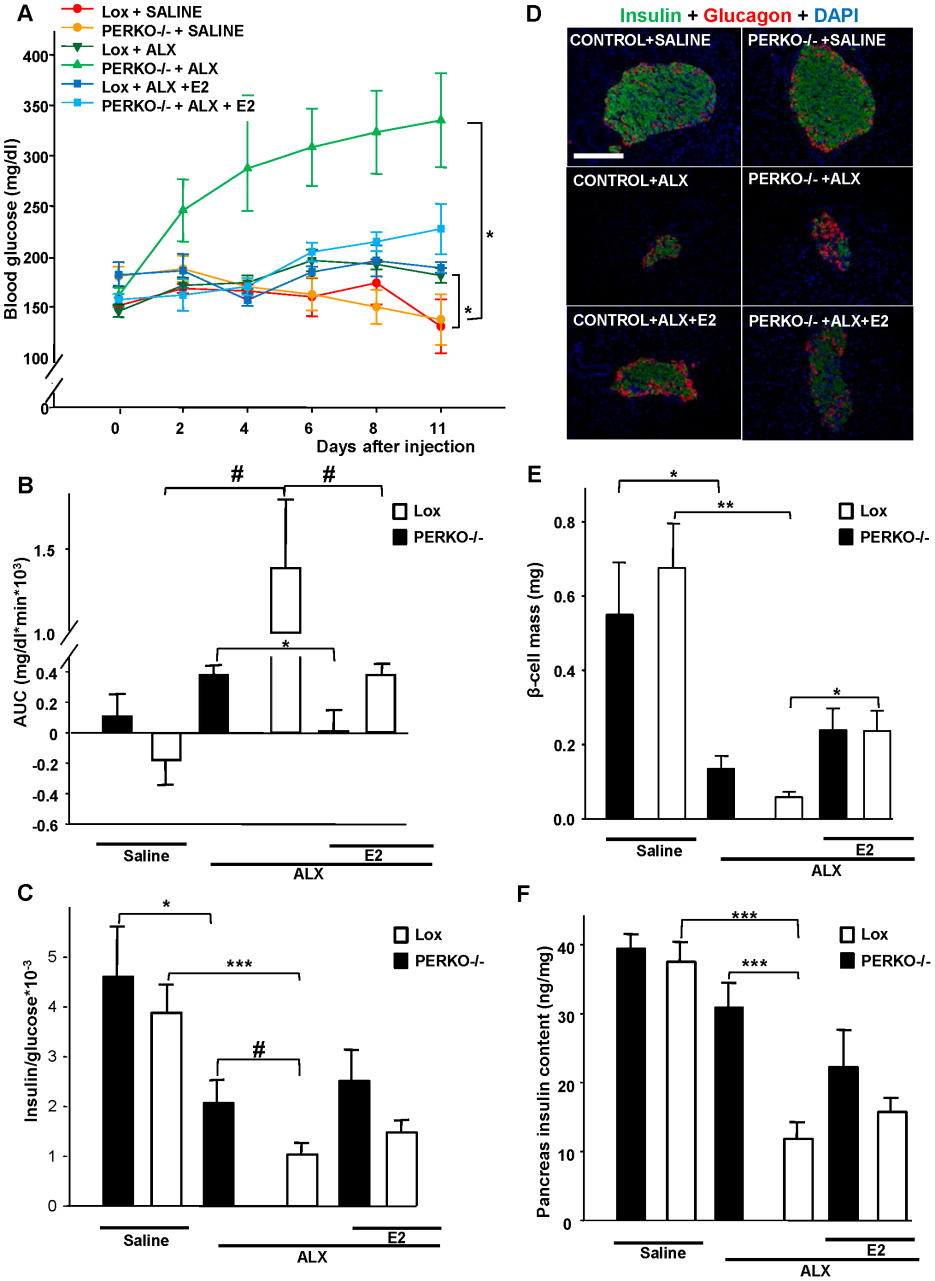
Male PERαKO^−/−^ mice are susceptible to ALX-induced diabetes. **(A)** Random-fed blood glucose from day 0 to day 11 after injection of either 150 mg/Kg of ALX or saline. **(B)** Corresponding area under the curve (AUC) for glucose. **(C)** Ratio of random-fed of insulin (ng/ml) and glucose (mg/dl) at day 11 was used as an index of insulin deficiency. **(D)** Representative sections showing immunofluorescent histochemical analysis of pancreas sections stained for insulin (green) and glucagon (red) in control ERαlox^+/+^ and PERαKO^−/−^ mice **(E)** β-cell mass quantification **(F)** Pancreas insulin concentration 11 day after ALX injection. Values represent the mean±SEM, n = 4–19/group. *P<0.05, ***P<0.001, #  = 0.06. Bar represents 100 µm.

Regarding males, control and PERαKO^−/−^ mice were normoglycemic and normoinsulinemic in basal conditions (Fig. 5A–C), and displayed normal islet architecture (Fig. 5D). After ALX exposure, both control and PERαKO^−/−^ male mice developed hyperglycemia and insulin deficiency and exhibited decreased β-cell mass and pancreatic insulin concentrations. However, the reduction in all of these parameters was more dramatic in PERαKO^−/−^ than in control mice (Fig. 5A–F). In addition, after E2 administration, we observed partial protection from alloxan-induced β-cell destruction and insulin deficiency in both controls and PERαKO^−/−^ male mice (Fig. 5A–F). Thus, as observed in females, male PERαKO^−/−^ mice exhibited a predisposition to alloxan-induced β-cell destruction (although to a lesser extent), but estrogen still provided some protection from alloxan in the absence of islet ERα. Note that unlike in the case of Fig.3, experiments of ALX injections described in Fig.4 and 5 were performed independently in male and female mice. Therefore, males and female mice described in in Fig.4 and 5 are not comparable with regard to the female protection from diabetes observed in Fig.2.

### Altered islet ERα expression during hyperglycemia and hyperinsulinemia

**Figure 6:**
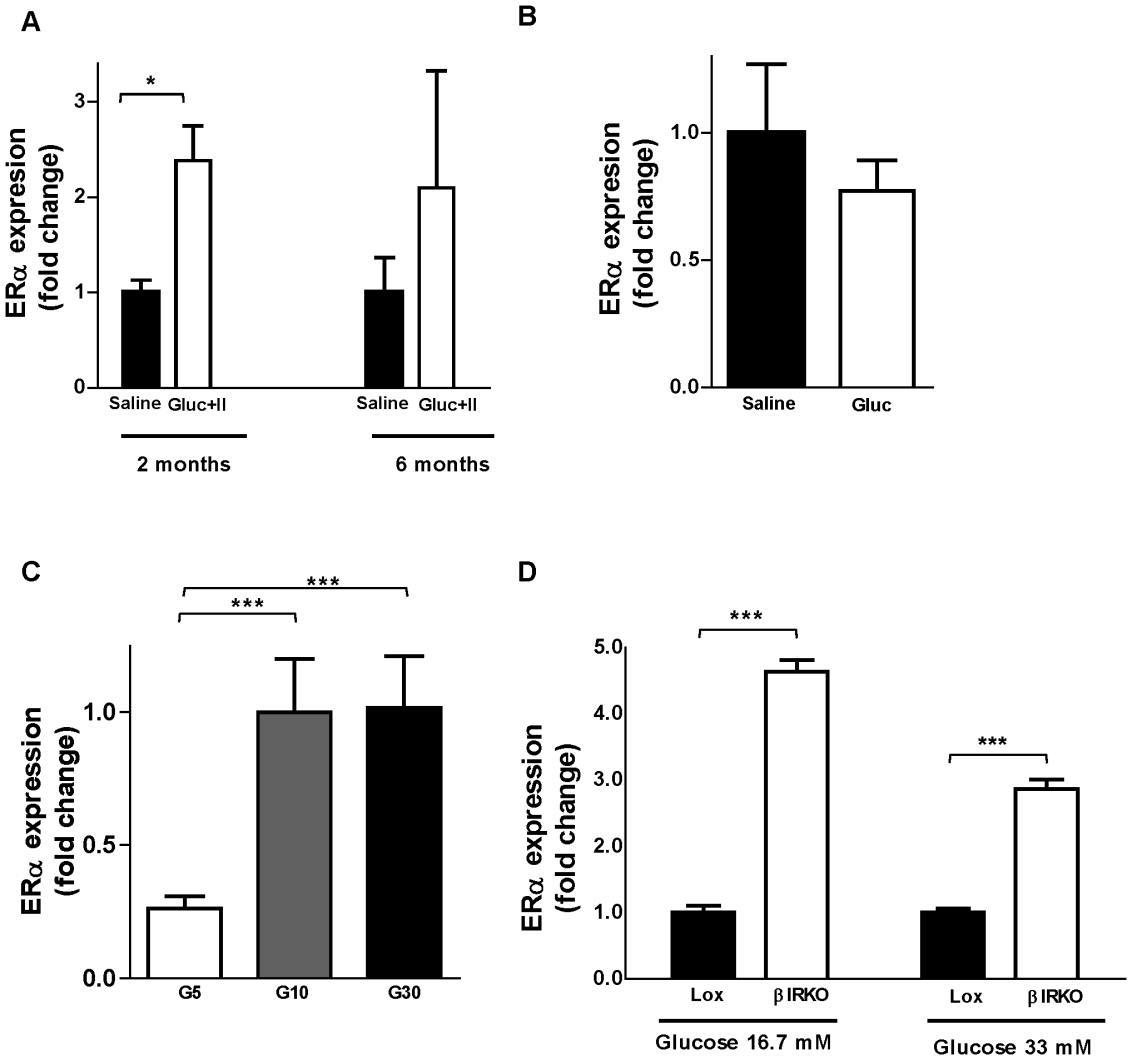
Messenger RNA levels of ERα were measured by QT-PCR from **(A)** 2 and 6 months-old male Wistar rats after 72 h glucose and intralipid co-infusion.**(B)** C57bl/6J male mice of 8–12 weeks-old after a 4 day glucose infusion. **(C)** Wistar rat islets cultured for one week in glucose 5, 10 or 30 mM. **(D)** β-cells cell line from control Lox/Lox and βIRKO mice cultured in 16.7 or 33 mM glucose. Values represent the mean±SEM, n = 4–5/group. *P<0.05, **P<0.01, ***P<0.001.

Having determined that islet ERα is important to oxidative stress protection *in vivo*, we next sought to determine whether ERα mRNA expression was altered in islets during hyperglycemia-induced oxidative stress *in vivo*. We used two established rodent models of glucotoxicity and glucolipotoxicity. We first studied ERα mRNA expression in islets from non-diabetic Wistar rats that received a 72 h glucose and intralipid co-infusion to mimic glucolipotoxicity (mean glucose 15 mM) [14]. Under these conditions, hyperglycemia was associated with increased ERα mRNA expression in 2 month-old rat islets (Fig. 6A). However, hyperglycemia did not increase ERα mRNA in islets from 6 month-old rats. We next studied ERα mRNA expression in a mouse model of mild hyperglycemia that was achieved by a 4-day glucose infusion (mean glucose 7 mM) [16]. In this model, we observed no increase in islet ERα mRNA (Fig. 6B). To ascertain whether ERα mRNA induction under severe hyperglycemic conditions resulted from a direct glucose effect on islets, we further studied ERα mRNA expression in Wistar rat islets cultured one week in hyperglycemic conditions [15]. ERα expression was increased when glucose was raised from 5 mM to 10 mM, but there was no further increase at 30 mM (Fig. 6C). Therefore, moderate to severe hyperglycemia [15] is associated with increased ERα mRNA expression *in vitro* and *in vivo* in rats.

### ERα expression in insulin resistant β-cells

We hypothesized that the increased ERα mRNA expression in islets exposed to hyperglycemia could be due to the stimulatory effect of high glucose or to the impact of elevated insulin on the IR in the islets. To address this question, we quantified ERα expression in β-cells isolated from normal and β-cell IR knockout (βIRKO) mice [18]. These islets were cultured in hyperglycemic conditions to increase insulin secretion. Consistent with the effect of glucose described above (Fig. 5A), ERα mRNA expression was increased in both control (lox/lox) and βIRKO β-cells when glucose was increased from 16.7 mM to 33 mM (Fig. 6D). However, at both glucose concentrations, ERα mRNA expression was higher in βIRKO compared to lox/lox β-cells, demonstrating that insulin action in β-cells inhibits ERα mRNA expression.

## Discussion

Having established that ERα is not essential to islet angiogenesis in mice, we focused on the role of ERα in protecting islets from glucotoxicity and oxidative stress *in vivo* and report that both male and female mice lacking ERα selectively in the pancreas are more susceptible to alloxan-induced β-cell destruction, insulin deficiency, and hyperglycemia. Although these experiments demonstrated a mild decrease in ERα mRNA expression in hypothalamus of PERαKO^−/−^ mice, the absence of alteration in energy homeostasis [7], [8] and the pancreas-specific phenotype observed in this model both suggest that the PERαKO^−/−^ phenotype results exclusively from pancreatic elimination of ERα. Since alloxan induces oxidative stress, these findings demonstrate that normal islet ERα expression is required to protect β-cells from oxidative stress-induced apoptosis *in vivo* in both sexes. The harmful effect of ERα deletion is more pronounced in female mice, presumably as a result of higher E2 serum concentrations that are required to activate the islet ERα in this gender. Nonetheless, the negative effect of ERα deletion on islet cells is also observed in males, demonstrating that islet protection by ERα is sex independent. We previously reported that mice of both sexes globally lacking ERα (αERKO^−/−^) were predisposed to streptozotocin-induced β-cell apoptosis and insulin-deficient diabetes [9]. However, the beneficial actions of estrogen on glucose homeostasis results from the combined actions of ERα in different tissues [2]. Thus αERKO^−/−^ mice globally lacking estrogen action in skeletal muscle, adipose tissue, and the brain become obese and insulin resistant as well as mildly hyperglycemic. This could produce additional β-cell stress that would synergize streptozotocin toxicity to alter β-cell survival. The current study demonstrates that loss of ERα selectively in islets – while ERα is normally expressed in other tissues – is sufficient to induce β-cell destruction in the presence of another β-cell stress and without any influence of altered body weight [2], [7]. Given the mild decrease in ERα expression observed in PERαKO^−/−^ hypothalamic ARC, this abnormality is unlikely to play a role in the PERαKO^−/−^ phenotype.

We previously reported that ERα gene dosage plays a role in the islet protection from streptozotocin injury because heterozygous αERKO^−/−^ mice of both sexes were predisposed to streptozotocin-induced diabetes [9]. Thus, increased ERα expression could function to protect β-cells against oxidative stress. To evaluate this hypothesis, we used established models of glucolipotoxicity and moderate hyperglycemia. We observed that in both cultured rat islets and in mouse islets *in vivo*, moderate to severe hyperglycemia increased ERα mRNA. In cultured mouse islets and MIN6 cells, short term exposure to high glucose is also associated with an increase in ERα mRNA expression [21]. Overexpression of ERα prevents apoptosis in PC12 neuronal cells, [22] and in the SK-N-MC human neuroblastoma cell line [23]. In contrast, in a model of moderate hyperglycemia, ERα mRNA is not upregulated in islets.

During hyperglycemia, is ERα induced by glucose itself or by insulin? In IR-deficient β-cells cultured in high glucose, ERα mRNA was increased compared to normal cells. This demonstrates that compared to hyperglycemia, insulin action in β-cells is unlikely to play a direct role in inducing ERα mRNA in β-cells. Thus, the induction of ERα expression by hyperglycemia could function as a β-cell protection against oxidative injury when hyperglycemia reaches a threshold beyond which oxidative injury occurs. Further, glucolipotoxicity, upregulates ERα mRNA in young rats, but this feature is lost in older animals. Since ERα improves β-cell survival [3], [5], [9], the loss of ERα induction in old islets may alter their resistance to diabetic injuries, as we observe in the PERαKO^−/−^ mouse. This weakness may further increase β-cell susceptibility to oxidative injuries such as glucotoxicity, setting the stage for β-cell failure in old age.

ERα protection from oxidative stress could involve a combination of rapid antiapoptotic actions that are independent of nuclear events and that potentially lead to alteration in protein phosphorylation [3], [24]. Alternatively, it could involve a more classical genomic mechanism that induces an anti-inflammatory cascade via expression of the liver receptor homolog [25].

In conclusion, ERα mRNA expression is induced in islets from young mice by exposure to hyperglycemia and oxidative stress, and mice of both sexes that selectively lack ERα in the islets are susceptible to both oxidative stress in β-cells and insulin-deficient diabetes.
